# Imaging of Scleral Collagen Deformation Using Combined Confocal Raman Microspectroscopy and Polarized Light Microscopy Techniques

**DOI:** 10.1371/journal.pone.0165520

**Published:** 2016-11-02

**Authors:** Nilay Chakraborty, Mian Wang, Jason Solocinski, Wonsuk Kim, Alan Argento

**Affiliations:** Department of Mechanical Engineering, University of Michigan, Dearborn, MI, 48128, United States of America; Queen Mary University of London, UNITED KINGDOM

## Abstract

This work presents an optospectroscopic characterization technique for soft tissue microstructure using site-matched confocal Raman microspectroscopy and polarized light microscopy. Using the technique, the microstructure of soft tissue samples is directly observed by polarized light microscopy during loading while spatially correlated spectroscopic information is extracted from the same plane, verifying the orientation and arrangement of the collagen fibers. Results show the response and orientation of the collagen fiber arrangement in its native state as well as during tensile and compressive loadings in a porcine sclera model. An example is also given showing how the data can be used with a finite element program to estimate the strain in individual collagen fibers. The measurements demonstrate features that indicate microstructural reorganization and damage of the sclera’s collagen fiber arrangement under loading. The site-matched confocal Raman microspectroscopic characterization of the tissue provides a qualitative measure to relate the change in fibrillar arrangement with possible chemical damage to the collagen microstructure. Tests and analyses presented here can potentially be used to determine the stress-strain behavior, and fiber reorganization of the collagen microstructure in soft tissue during viscoelastic response.

## Introduction

Collagen fibers are the main constituent of the extracellular matrix of most soft tissues. In this work, an opto-mechanical characterization technique for collagenous tissue is demonstrated using scleral tissue as a model. Sclera is a complex tissue composed predominantly of water (72.2%) and collagen (22.1%) along with trace amounts of mucoid (2.3%), elastin (1.4%) and other proteinous constituents [1]. It is layered tissue and its underlying collagen microstructure has been found to vary greatly in specific fibrillar arrangement from region to region of the globe [2, 3]. Although the assembly and structure of collagen fibrils in sclera has been well-characterized, knowledge of its physico-chemical properties during mechanical loading is limited. Characterization of microstructural behavior and related mechanical responses in relation to the change of chemical properties of the extracellular matrix can improve the understanding of the physico-clinical impact of degenerative eye diseases that involve increased intraocular pressure. This characterization is also important to understand how the eye responds to and manages external insults such as projectiles, shocks, airbags as well as more nominal insults such as rubbing [4–7]. Though eye tissue is chosen as a model in this work, the method is equally applicable to any biological soft tissue such as ligaments, arteries and heart tissue.

Microscopy has been used extensively to image the un-deformed microstructure of tissue for anatomical or diagnostic purposes. A few of the more recent studies of this type specifically concerned with eye tissue are [2, 8–12]. Imaging tissue microstructure that is under load and deforming is more difficult and fewer works are available. Tissue constructs, artificial tissue, and other biomaterials are used as scaffolds, tissue surrogates, or purely for research purposes. Some work [13–15] is available on the imaging and modeling of their microstructure under load and deformation. Studies on the imaging of deformed or deforming real soft tissue microstructure are given in [16–19] and in [20, 21] for eye tissue. Specifically, the biaxial stretch of porcine and bovine heart tissue was imaged using small angle light scattering in [16] and found to produce nonaffine deformation. This was also found to be the case for the collagen gel treated in [13]. That the tissue deforms in a nonaffine manner complicates the use of simple analytical methods for determining the microscopic behavior of the tissue during global deformation, and demands the use of image-based techniques. Collagen recruitment during loading was studied for rabbit arteries in [17] using multi-photon imaging. Confocal microscopy was used to track tendon microstructural strain via photobleached grids in [18] and cell nuclei in [19]. Only a few works are available in which microstructural deformations of eye tissue are measured. In [20] an ultrasound elasticity microscope was used in conjunction with finite element methods to determine corneal strain. Corneal mechanical strain mappings were determined in [21] using polarized light microscopy, with a view toward an in-vivo method. The method produces strain fields in the overall cornea based on luminescence of strained collagen.

In recent years, the Raman spectroscopy technique has become an active area of research due to its ability to characterize biomolecules in their native state. Principles of Raman spectroscopy have been utilized in [22] to analyze microstructural response of mechanically loaded bone and bone tissue constructs in terms of global strain. Fourier transform infrared Raman microscopy was used to investigate molecular changes of collagen in human skin tissue under strain [23]. Even though the inherent limitations of the Fourier transform based Raman technique prevented the spectral information to be collected in a confocal plane, the study determines Raman signature in terms of global tissue strain. In [24], the molecular change in excised collagen fibers has been demonstrated by the shift in Raman wavenumbers before and after exposure to mechanical loading in a tensile test of the fiber. These studies provide significant evidence that strain can be an important driver of chemical changes in collagen fibers. Determining the associated changes in microstructural characteristics has a strong bearing on understanding existing disease models in collagenous tissues.

In this study a combined polarized light (PLM) and confocal Raman microscopy (CRM) technique is used to visualize the microscopic physico-chemical response of collagen microstructure and characterize microstructural strain in tissue as well as chemical denaturation that can result from the strain. Leveraging the optical anisotropic character of collagen fibers, PLM is used to directly observe the fibers in tissue samples while CRM is used to extract spatially correlated spectroscopic information in the same confocal plane. In this way, a two-dimensional chemical image of the biological tissue can also be constructed (hyperspectral imaging). The method is demonstrated using porcine scleral tissue which serves a structural function in the eye to resist intraocular pressure, muscle forces, insults, and other loads. As in other structural tissues, globally observed response of the sclera is a product of the biomechanical and biochemical behaviors of the underlying microstructure that can be revealed using the presented methods. Results are given showing the response and orientation of the collagen network of sclera in its native state as well as during global tensile and compressive loadings. An example is also given showing how the data can be used with a finite element program to estimate the strain variation in individual collagen fibers.

## Materials and Methods

### Specimen Preparation

Porcine eyes have been chosen for this initial study though the described test method and analyses are applicable to human eyes. Porcine eyes are similar to human eyes in geometry, behavior and normal intraocular pressure level [25] so they are often used in precursor and model studies [26] for human eyes. Intact porcine globes were obtained from local slaughterhouses (Scholl's Slaughterhouse, Blissfield, MI and Milligan's Northwest Market, Jackson, MI). Initial dissection, preparation, and handling of globes followed the procedure used in [27] and outlined here. Briefly, after removing orbital fat and muscle from the globe, the optic nerve was trimmed flush to the scleral surface. For the cut specimen tests, the globe was opened and the internal contents removed leaving a scleral “shell.” Rectangular specimens were hand-cut from the prepared sclera having size of roughly 2cm X 2cm. Circular specimens were stamped from larger pieces of sclera using an 8mm punch. These were used for compression tests. For tensile tests, 8mm X 8mm specimens were stamped from the rough cut pieces using a rectangular punch. Following [28], all tissues were kept hydrated in sealed containers containing gauze moistened with ophthalmic balanced salt solution (Alcon Laboratories Inc, Fort Worth, TX). As was found in [27], this maintains humidity at about 95%, as monitored by a humidity probe inserted in the container. The tissues were re-moistened frequently throughout dissection and specimen cutting procedures. Before testing, the tissue samples were rehydrated using Dulbecco's Modified Eagle's Medium and 1% penicillin-streptomycin for 10 mins followed by incubation in 0.25% trypsin for 5 mins. The tissue sample was washed 3 times using isotonic saline solution before application of mechanical loading and imaging described in the following section.

A goal of this work is to demonstrate a method for visualizing the movement of collagen fibers in tissue. To induce microstructural deformation, compressive or tensile loads are applied to the tissue specimens and measurements are made of the microstructure at an arbitrary point of the larger piece of tissue. No mathematical connection between the physical loads and the deformation is conducted. The tests have been designed to induce deformation in the tissue using global compression and tension of specimens so that the microstructure can be imaged during deformation.

### Test Set-Up and Mechanical Loading

**Fig 1:**
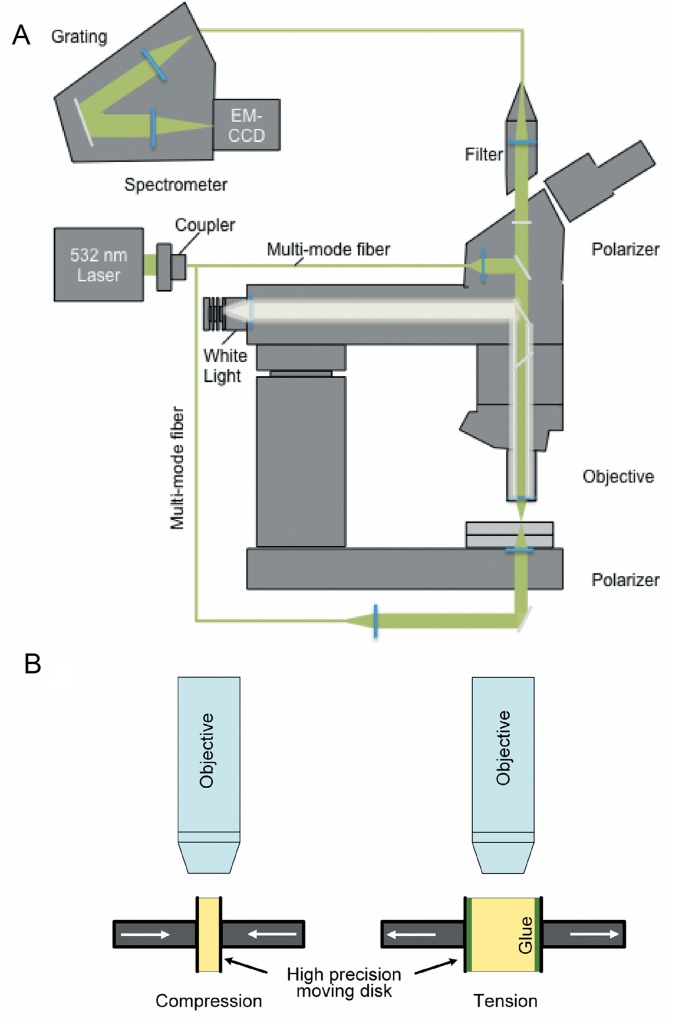
(A) Block diagram of the measurement system. (B) Schematic showing test specimens loaded in compression and tension on the measurement stage.

Fig 1A shows a block diagram of the measurement system that will be described in detail later. Specimens were imaged simultaneously by PLM and CRM. For the whole globe test, the globe was placed on the stage in a shallow cup containing moistened gauze. Two types of cut specimen tests were conducted with the specimens fixed in grips on the measurement stage as shown schematically in Fig 1B. In the first type, global compression was induced in the 8mm circular specimens. These were placed between the measurement heads of a digital micrometer (H-2780, Mitutoyo Corp.) through which global compressive displacement was applied in 0.02mm increments. In the second test, tensile loading was applied to one pair of parallel edges of the rectangular specimens. These edges were glued to the same micrometer and tensile displacement was applied in 0.02 mm increments. In the compressive tests, the imaging site was on the outer cut edge of the specimen. In the tensile tests, a location was selected near the center of a cut edge of the specimen, far from the glued edges. No evidence of glue was apparent at the imaged points.

### Microscopic Imaging

Two different microscopy techniques were used in the investigation of the microstructural deformation of the collagen network in tissue–polarized light microscopy and spatially correlated confocal Raman microscopy. The imaging system is shown in the diagram, Fig 1A. A relatively simple PLM setup was used to perform the polarized light microscopy of decellularized porcine sclera. Linear PLM was performed on a modified upright Zeiss Microscope (AxioExaminer, Carl Zeiss, Oberkochen, Germany) with the addition of a polarizer and analyzer. The polarizer was placed after the light source, which ensured that only the linearly polarized light perpendicular to the direction of light propagation was transmitted to the tissue specimen. Fibrillar structures in tissue split incident polarized light into two orthogonal rays. The analyzer filter, positioned after the specimen and at a right angle to the polarizer, recombines rays split by collagen to create the observed image. The orientation of the analyzer ensures that only light with a polarization that was altered by the tissue is transmitted. The intensity of the resulting signal therefore indicates the regions of the tissue that are optically active, or in other terms, birefringent, anisotropic, or oriented. PLM images of deforming microstructure were captured using a high speed charge coupled device camera (iDus 420, Andor, Belfast, UK).

**Fig 2:**
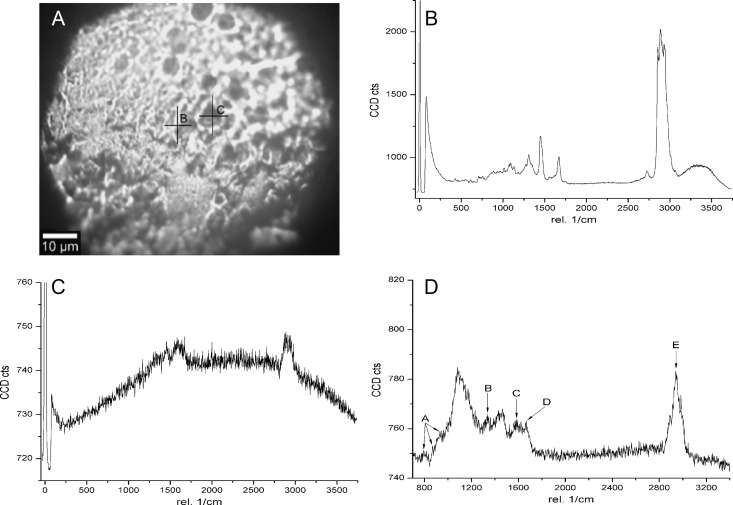
(A) PLM image of porcine optic nerve head tissue roughly flush with the globe’s surface. Raman scan of two points (B) and (C) having distinctly different visual features shown as cross-marks on the image. Fig (D) Indicates the Raman spectra for Collagen I showing the following characteristic peaks: A = three characteristic peaks related to pyrrolidine ring of proline backbone of collagen. (~855 cm^-1^), B = Amide III (~1320 cm^-1^), C = Amide II (~1490 cm^-1^), D = Amide I (~1640 cm^-1^) and E = C-H stretch (~2900 cm^-1^).

A pinhole based confocal Raman microscopy setup [29] was used to collect microspectroscopic information from the deformed/un-deformed collagen network within the tissue. Spectral information was acquired at each point from a specific X-Y plane of the tissue and was collected using a 532nm solid-state visible laser and a servo scanning stage (Fig 1A). A two-dimensional chemical composition map of the molecule of interest at each point of the plane was generated by integration of the selected bands representing the molecule of interest at each point. A 10x Zeiss objective was used to capture 10,000 Raman signals from a window of 50×50μm^2^. CRM measurements were used as endpoint studies before and after compressive loading to elucidate the change in the principal chemical composition of the scleral fibers. By comparing the characteristic spectral signatures of collagen I with the spectroscopic signatures obtained from porcine sclera, an understanding of the spatial distribution of collagen I was developed. Collagen I network in both un-deformed and deformed porcine sclera were acquired by generating hyperspectral images against characteristic collagen peaks. Reference Raman spectral signatures determined using standard collagen solutions (Sigma Aldrich, St. Louis, MO; see for example Fig 2D, which will be discussed in detail later). Collagen I, in part, can be identified from spectral signatures of some of its chemical constituents. For collagen I, the characteristic C–C bond around the wavenumber 855cm^−1^, representing the pyrrolidine ring of proline backbone [30], was used for identification. In addition, the collagen I signature was verified by the position of the amide I band, which is centered at 1640 cm^−1^ in the type I collagen.

## Results and Discussion

Fig 2 shows the characteristic PLM image of porcine optic nerve head tissue in an intact whole globe. The optic nerve was snipped nearly flush with the globe’s outer surface revealing an internal tissue. Imaging was directed normal to the globe at a specific point on this tissue. Point-scans of Raman spectra at locations (B) and (C) on Fig 2A are given in Fig 2B and 2C. In addition, the Fig 2D indicates the characteristic Raman spectra for pure type I collagen. The characteristic C–C stretch is visible around the wavenumber 855cm^−1^ representing the pyrrolidine ring of the proline backbone [30] in collagen. Consistent with existing literature [30], the amide I band is found to be centered at 1640 cm^−1^ arising from the C = O stretching of the peptidic bond in the GlyX-Y tripeptide sequence which is the proteinous component of collagen I. All of these characteristics peaks (discussed in detail in the figure legend) in Fig 2D are visible in the Raman spectral scan at location (B). This indicates that Point (B) likely lies on a collagen containing tissue whereas Point (C) lies on tissue with much less collagen.

**Fig 3:**
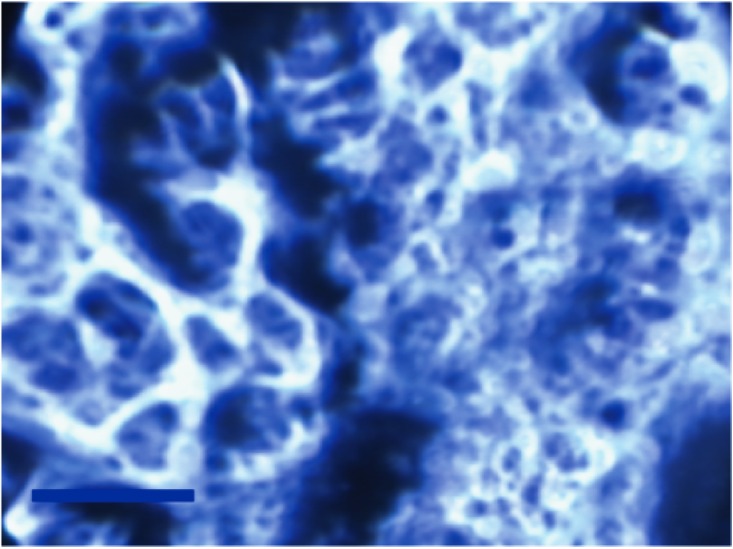
Polarized light microscopy of a sample of porcine scleral tissue near the optic nerve head. The figure shows the intricate network of the collagen fibers in the tissue in the un-deformed condition. The scale bar in the image indicates 10 μm.

In this study, the PLM technique exploits the optical properties of fibrillar collagen that changes the direction of polarized light—an effect known as birefringence. Here, it was confirmed that the collagen in scleral tissue splits the incident polarized light into two orthogonal rays in a way that depends on the orientation of collagen at each point in the section. As shown in the micrograph, Fig 3, of un-deformed porcine scleral tissue cut from a location immediately adjacent to the optic nerve head, the collagen orientation can be inferred from the presence of the birefringent (white) versus non-birefringent (dark) regions. The image indicates the intricate framework of the collagen organization in sclera that is crucial for its ability to resist the intraocular pressure, muscle forces that produce globe movements, and external insults without excessive deformation so that sight can be maintained in the presence of the loads. It should be noted that although here the tissue is described as “un-deformed,” sclera in its native state is normally subjected to in-plane tension and out-of-plane compression from the intraocular pressure.

**Fig 4:**
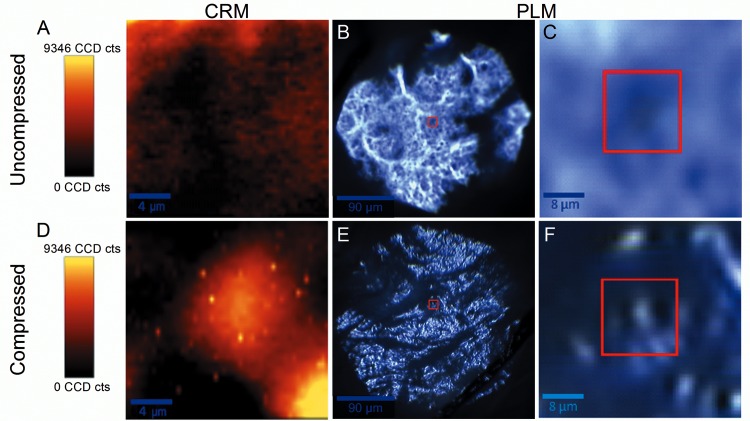
Hyperspectral chemical images of the collagen network of porcine scleral tissue in un-deformed (A) and deformed (D) states generated by the spatially correlated CRM imaging technique. The figure also shows the corresponding PLM images (B, C, E and F) of the same tissue. The horizontal direction in each image corresponds to the direction in which deformation is applied to the overall sample.

**Fig 5:**
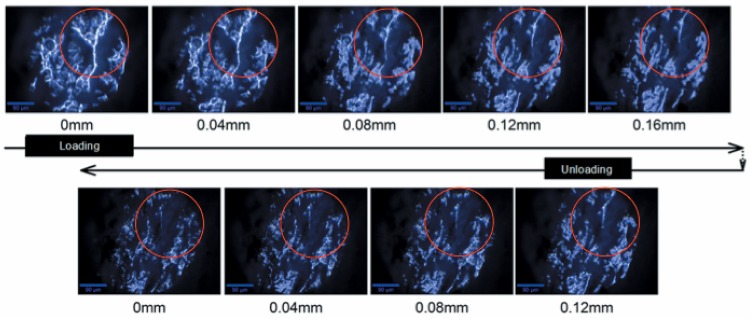
PLM of porcine sclera microstructure under global sequential compression of a tissue sample followed by unloading. Numbers under the images indicate global deformation of the tissue sample. The horizontal direction in each image corresponds to the direction in which deformation is applied to the overall sample. The scale bar is 90 μm.

The combined PLM and CRM imaging approach permits tissue microstructural imaging while the tissue deforms allowing study of how the collagen microstructure rearranges itself under load. In Fig 4, the spatially correlated CRM imaging technique was used to generate hyperspectral chemical images of the collagen network of scleral tissue in un-deformed (Fig 4A) and highly deformed (Fig 4D) states. Here compressive loading was applied to the tissue sample as shown in Fig 1B. The figure also shows the corresponding PLM images (Fig 4B, 4C, 4E and 4F) of the same tissue. The PLM image of the un-deformed collagen network is given in Fig 4B and the hyperspectral chemical image of a smaller region of the same scleral tissue, identified by the red square in Fig 4B and 4C, is shown in Fig 4A. Under compressive load, the change in collagen fiber morphology is demonstrated by the PLM image in Fig 4E compared to that in Fig 4B. Here the overall tissue sample was deformed in compression to a high average strain level (~11%) causing large disruption of the collagen network. The overall movement of the scleral network was tracked using the change in micrographic images (described in the progressive loading of scleral tissue as seen in Fig 5). Geometric and structural correlation can be seen between the collagen fibers imaged using PLM and the hyperspectral images created using CRM. For example, the small region of tissue identified by the red square in Fig 4E is enlarged in Fig 4F and one can see, despite some blurring due to limitations of the numerical aperture of the lens assembly itself, a distribution of collagen (white) mirrored by the chemical signature for collagen in Fig 4D (yellow). In contrast, the tissue in the red square prior to loading (Fig 4C) lacks substantial collagen content as indicated by the dark region in Fig 4A. It is important to note that this significant change in the hyperspectral image of the collagen network indicates a change in the tissue constitution at the imaged point as the tissue deforms. Here collagen moves into the region identified by the red square. Also, under the global compressive load, the collagen microstructure appears to have been significantly altered as seen by the highly concentrated spots of collagen in Fig 4D.

A way in which progressive loading can impact collagen microstructure in tissue is illustrated in Fig 5. A sequence of PLM images was acquired during compressive deformation of an 8mm diameter X 1.5mm (roughly) thick sample of porcine scleral tissue as demonstrated in Fig 1B. Note that the specimen is oriented such that the loading is directed parallel to the through-the-thickness direction of the scleral wall resulting in out-of-plane compression. In an actual globe, the intraocular pressure induces out-of-plane compression in the sclera by its direct bearing on the inner wall of the globe and indirectly through lateral contraction that results from the induced in-plane tension in the sclera. Compression can also occur in sclera or cornea from external insults to the eye such as projectile impact or shock waves, the latter of which will produce a compression wave in the deformable tissue [31]. During the test, compressive deformation is applied in 0.02 mm increments. After a global displacement of 0.16 mm, the global compression was reduced following the same 0.02 mm increments. Significant re-orientation of the fibrillar structure in the tissue is seen to progressively occur in Fig 5 as the global deformation is increased. For example, this is readily apparent in the circled grouping of collagen that can be seen to undergo substantial movement in the image sequence. This example illustrates measurements of deforming microstructure during global strains up to about 10%. It is interesting to note that even after the global compression is completely removed, the tissue microstructure does not immediately regain its initial morphology as seen by comparing the first image in row 1 to the first image in row 2. It was not determined in the present study whether or not the microstructure underwent permanent damage or if it would gradually return to its initial morphology through a viscoelastic response, though observation of creep and relaxation of microstructure could be made using the present method. Macroscopic viscoelastic behavior of ocular tissues has been observed in a number of studies, for example [32–34]. How the response of the microstructure leads to the macroscopically observed viscoelastic phenomena is unknown at present.

**Fig 6:**
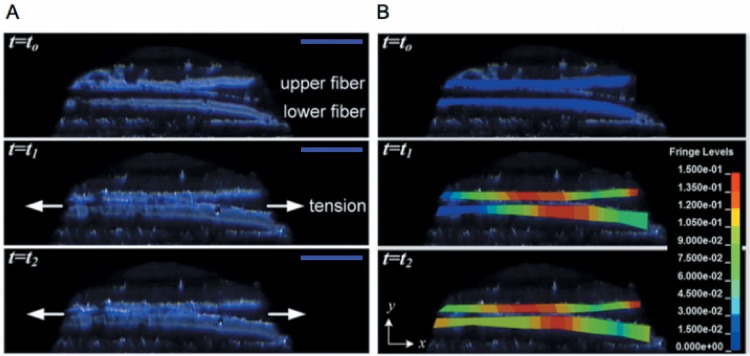
Collagen fibers in a sample of porcine sclera under tensile loading. (A) PLM images and (B) calculated normal strain *ϵ_xx_*. The arrows indicate the direction in which tensile deformation is applied to the overall 8mm X 8mm sample. The scale bars in the figure(s) represent 90 *μ*m.

The most prevalent mode of loading in sclera is in-plane tension due to the intraocular pressure. Fig 6A shows a series of PLM images of a deforming microstructure in which a few individual collagen fibers are identified. Here a tensile deformation was applied to an 8mm X 8mm tissue sample in 0.02mm increments as shown in Fig 1B. A microstructural region of the tissue was imaged as the load was increased. The top image of Fig 6A shows reference status and the second and third row images display increasingly deformed status. It can be seen that two separately located fibers at the initial time *t* = *t*_0_ are becoming merged under tensile load at *t* = *t*_2_. It is also seen that more fibers from underneath the image plane become engaged as tension increases and there appears to be an agglomeration of fibers at the bottom of the *t*_1_ and *t*_2_ images.

Displacement and deformation of fibers in Fig 6A were quantified to determine strain by tracking the coordinates of points on the fibers in the series of PLM images. As an example, 29 points on these fibers were tracked and normal strain, *ϵ_xx_*, of the deforming fibers calculated as shown in Fig 6B, neglecting out-of-plane motion. Here the strain field has been computed using the true strain model in the finite element program LS-DYNA by importing the tracked coordinates of the selected points over the time *t* = *t*_0_ to *t* = *t*_2_. Fig 6B shows that there is a spatial variation of strain in the fibers, and the normal strain is generally greater in the central region of the fibers in the presented case. As the upper fiber nears the lower fiber at *t* = *t*_1_ and *t* = *t*_2_, the normal strain gradient in the lower fiber is seen to become more uniform, suggesting how stress and strain fields in the tissue are effected by interaction of the fibers. For a large scale problem, standard particle tracking software such as Image J (National Institute of Health, Bethesda, MD) [35] or Metamorph (Universal Imaging Corp., West Chester, PA) [36] can be used to track points for subsequent strain calculations. A marker-less image tracking and correlation technique was demonstrated in [37] for collagen fiber deformation that is applicable to track tissue points in a large number of sequential PLM images generated by the present method.

Some important studies are feasible using the techniques described here. A finite element program has been used as a convenient way to determine the normal strain and shear strain (not shown here) from the measured displacement fields, and to graphically present it. If the material properties of the collagen fibers and surrounding tissue are estimated, a microstructural model can be constructed to calculate the resulting stress fields in the anisotropic microstructure allowing investigation of the interaction between the fibers and the surrounding soft tissues as well as the attendant load transfer. Simultaneously monitoring collagen using PLM and CRM will also indicate what collagens are present in the tissue and whether their chemical nature change under severe loading. While full-field optical coherence tomography has recently been used to perform elastographic contrast studies of tissue [38], a combination of PLM and CRM provides a comprehensive platform for analysis of deformation and chemical characterization of tissue. It is important to note that because of the preferentially forward-directed signal, trans-collection is the principal detection mode in Raman scattering. While it is possible to collect deep tissue Raman signals [39], epicollection is the preferred method of analysis due to limitations related to penetration of light [40]. Thus, equivalent two-photon fluorescence techniques can be used to study the effects of mechanical loading on microstructure in thicker tissue sections.

## Conclusions

The study conducted here provides a new imaging tool to simultaneously characterize deformation and related chemical changes in collagenous tissue using polarized light and confocal Raman microscopies. Porcine sclera has been used to demonstrate the technique. The capability of the method to both reveal microstructural features and chemically differentiate them has been demonstrated. The results show movement patterns of the collagen microstructure while a larger sample of tissue is loaded and unloaded. The study indicates a number of interesting features that could be attributed to microstructural damage, viscoelasticity, and collagen reorganization. Further investigation to elaborate these findings could shed light on the relationships between a tissue’s microstructural and global behaviors. This optospectroscopic characterization technique could benefit from some improvements including using an electron multiplying charged coupled camera to increase image sensitivity and a piezo-electric fixture mechanism to refine the mechanical loading methods. This work has application to characterizing the response of tissues to loading in healthy and diseased states, and so can be used to help understand progression of diseases related to tissue failure. Relevant tissues include arteries, tendons, ligaments, abdominal wall tissues and cornea, as well as sclera used to demonstrate the methods here. In particular, when applied to human eyes, the work has implication to understand the response of scleral microstructure in the vicinity of the optic nerve where cellular damage occurs in glaucoma.
